# Recent developments in mushrooms as anti-cancer therapeutics: a review

**DOI:** 10.1007/s13205-011-0036-2

**Published:** 2011-11-25

**Authors:** Seema Patel, Arun Goyal

**Affiliations:** 1 Department of Biotechnology, Lovely Professional University, Jalandhar, 144402 Punjab India; 2 Department of Biotechnology, Indian Institute of Technology Guwahati, Guwahati, 781039 Assam India

**Keywords:** Polysaccharides, β-Glucan, Anti-tumor agent, Apoptosis, Caspase

## Abstract

From time immemorial, mushrooms have been valued by humankind as a culinary wonder and folk medicine in Oriental practice. The last decade has witnessed the overwhelming interest of western research fraternity in pharmaceutical potential of mushrooms. The chief medicinal uses of mushrooms discovered so far are as anti-oxidant, anti-diabetic, hypocholesterolemic, anti-tumor, anti-cancer, immunomodulatory, anti-allergic, nephroprotective, and anti-microbial agents. The mushrooms credited with success against cancer belong to the genus *Phellinus*, *Pleurotus*, *Agaricus*, *Ganoderma*, *Clitocybe*, *Antrodia*, *Trametes*, *Cordyceps*, *Xerocomus*, *Calvatia*, *Schizophyllum*, *Flammulina*, *Suillus*, *Inonotus*, *Inocybe*, *Funlia*, *Lactarius*, *Albatrellus*, *Russula*, and *Fomes*. The anti-cancer compounds play crucial role as reactive oxygen species inducer, mitotic kinase inhibitor, anti-mitotic, angiogenesis inhibitor, topoisomerase inhibitor, leading to apoptosis, and eventually checking cancer proliferation. The present review updates the recent findings on the pharmacologically active compounds, their anti-tumor potential, and underlying mechanism of biological action in order to raise awareness for further investigations to develop cancer therapeutics from mushrooms. The mounting evidences from various research groups across the globe, regarding anti-tumor application of mushroom extracts unarguably make it a fast-track research area worth mass attention.

## Introduction

Mushrooms have been regarded as gourmet cuisine across the globe since antiquity for their unique taste and subtle flavor. Recently, it has been discovered that many mushroom species are miniature pharmaceutical factories producing hundreds of novel constituents with miraculous biological properties. They have a long history of use in Oriental medicine, but their legendary effects in promotion of good health and vitality are being supported by contemporary studies only. Of late, mushrooms have emerged as wonderful source of nutraceuticals, anti-oxidants, anti-cancer, prebiotic, immunomodulating, anti-inflammatory, cardiovascular, anti-microbial, and anti-diabetic (Barros et al. 2007; Sarikurkcu et al. 2008; Wang et al. 2004; Kim et al. 2007; Synytsya et al. 2009). The ongoing research projects are aimed to promote mushrooms as new generation “biotherapeutics”.

**Fig. 1:**
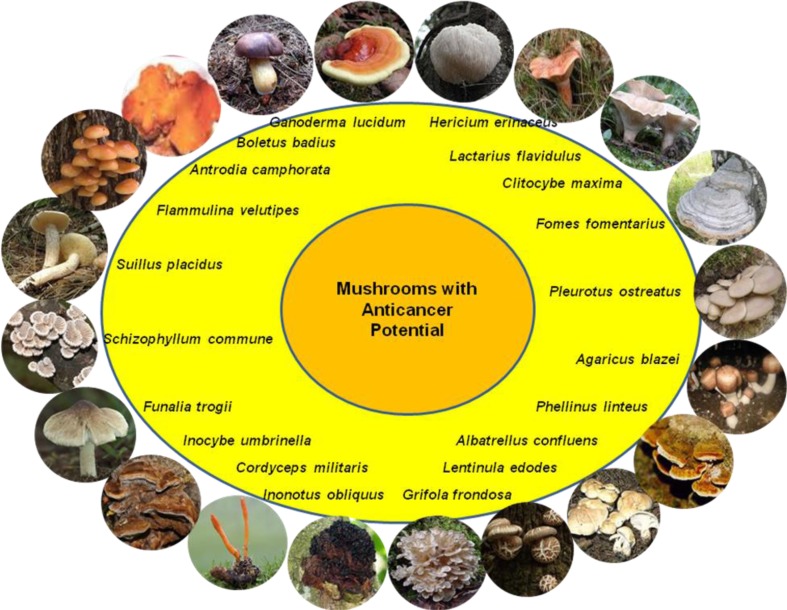
Some medicinal mushrooms with anti-cancer potential

**Fig. 2:**
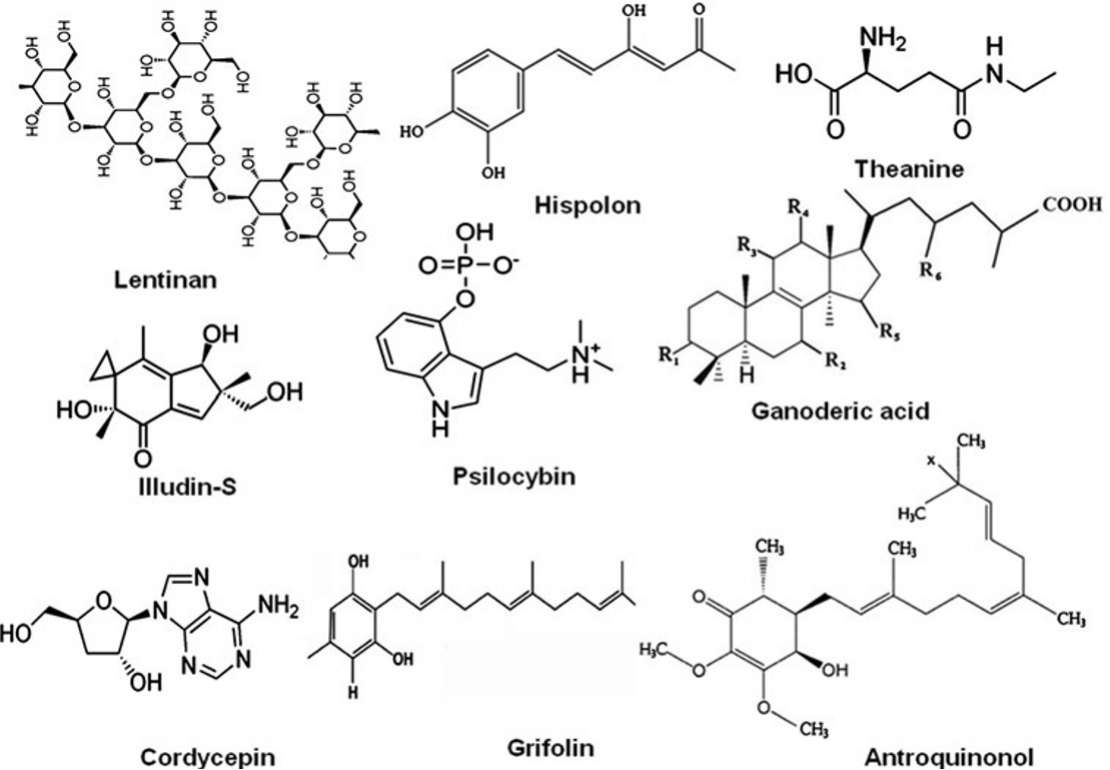
Structure of anti-cancer compounds isolated form mushrooms (collected from http://www.wikipedia.org)

**Fig. 3:**
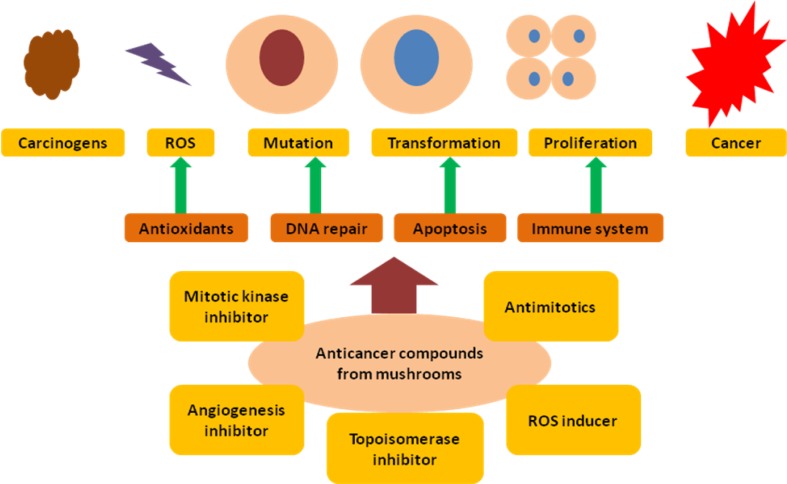
Anti-cancer mechanism of mushroom bioactive compounds

Cancer is a leading cause of death worldwide. The current anti-cancer drugs available in market are not target specific and pose several side-effects and complications in clinical management of various forms of cancer, which highlights the urgent need for novel effective and less-toxic therapeutic approaches. In this context, some prized mushrooms with validated anti-cancer properties and their active compounds are of immense interest. Numerous clinical trials have been conducted to assess the benefits of using commercial preparations containing medicinal mushroom extracts in cancer therapy. Their potential uses individually and as adjuncts to cancer therapy have emerged. Mushrooms are known to complement chemotherapy and radiation therapy by countering the side-effects of cancer, such as nausea, bone marrow suppression, anemia, and lowered resistance. Recently, a number of bioactive molecules, including anti-tumor agents, have been identified from various mushrooms (Fig. 1). The bioactive compounds of mushrooms include polysaccharides, proteins, fats, ash, glycosides, alkaloids, volatile oils, tocopherols, phenolics, flavonoids, carotenoids, folates, ascorbic acid enzymes, and organic acids. The active components in mushrooms responsible for conferring anti-cancer potential are lentinan, krestin, hispolon, lectin, calcaelin, illudin S, psilocybin, *Hericium* polysaccharide A and B (HPA and HPB), ganoderic acid, schizophyllan, laccase, etc. (Fig. 2). Polysaccharides are the best known and most potent mushroom-derived substances with anti-tumor and immunomodulating properties. The polysaccharide, β-glucan is the most versatile metabolite due to its broad spectrum biological activity. These β-glucans consist of a backbone of glucose residues linked by β-(1 → 3)-glycosidic bonds, often with attached side-chain glucose residues joined by β-(1 → 6) linkages (Chen and Seviour 2007). Their mechanisms of action involve their being recognized as non-self molecules, so the immune system is stimulated by their presence. Hispolon, an active polyphenol compound, is known to possess potent anti-neoplastic properties and potentiate the cytotoxicity of chemotherapeutic agents. The scientific investigations to back the claims have gained momentum in recent years. Findings suggest that some mushrooms in combination with commercial anti-cancer drugs work in synergy as an effective tool for treating drug-resistant cancers. In this study, the mechanisms underlying apoptosis induced by medicinal mushrooms are summarized (Fig. 3). This compilation is expected to provide new insights into the possible therapeutic use of the mushroom extracts against different cancers. These results are significant in that they provide a mechanistic framework for further exploration of the use of bioactive compounds as novel anti-tumor agents. The purpose of the present review is to summarize the available information and to reflect the current status of this research area with a view for future direction.

## Anti-cancer uses of mushrooms

### Genus *Phellinus*

*Phellinus* is a genus of mushrooms belonging to the family Hymenochaetaceae. *Phellinuslinteus* has anti-tumor, immunomodulating and anti-metastasis properties owing to its β-(1 → 3) linked glycan (Baker et al. 2008). The extract of *P*. *linteus* is reported to contain antimutaganic activities and play a role in the prevention of cancer by inducing NAD(P)H:quinone oxidoreductase and glutathione *S*-transferase activities. Hispolon, the phenolic compound extracted from this mushroom has potential to induce apoptosis of breast- and bladder-cancer cell (Lu et al. 2009). A protein-bound polysaccharide from this mushroom induces G2/M phase arrest and apoptosis in SW480 human colon cancer cells (Li et al. 2004). It has also anti-inflammatory and anti-angiogenic activities (Kim et al. 2004a). *Phellinuslinteus* methanol extract and its fractions, viz., methylene chloride, ethyl acetate, and *n*-butanol, have the potential for anti-angiogenic effects through the inhibition of human umbilical vein endothelial cells (HUVECs) proliferation, migration and assembly into capillary-like structures as well as in vivo angiogenesis. These findings indicate the potential for the use of the mushroom extract in stimulated angiogenesis, such as inflammation and tumor development (Lee et al. 2010). Huang et al. (2011) evaluated the anti-cancer effect of a mycelial culture of *P*. *linteus* and elucidated its potential mechanism in vivo. Human hepatoma (Hep3B) cell-transplanted mice when administered the mushroom extract daily for 8 weeks, a significant reduction in tumor size and increase in T cell numbers; IL-12, IFN-γ and TNF-α secretion; NK cell activity and phagocytic ability were observed. Therefore, increased numbers of CD4^+^ cells could have been caused by greater numbers of dendritic cells and macrophages in the spleen. Furthermore, the activation of dendritic cells and macrophages resulted in increased IL-12 secretion, which could up-regulate NK cell activation. Thus, *P*. *linteus* extract may provide a potential therapeutic approach for both immunomodulatory and anti-tumor effects. Li et al. (2011) studied the heavily glycosylated protein, proteoglycan purified from *P*. *linteus* to determine its possible anti-tumor effect on human cancer cells and mechanisms involved. Cell inhibition assay showed that proteoglycan has an anti-proliferative effect on human hepatocellular liver carcinoma (HepG2), Human colon adenocarcinoma (HT-29), human lung cancer (NCI-H 460) and human breast adenocarcinoma (MCF-7) cells. When HT-29-bearing mice were treated with 100 mg/kg proteoglycan, there was relative increase in spleen and thymus weights, the plasmatic immunoglobulin receptor pIgR and IgA levels were significantly increased. Measurement by ELISA showed a notable decrease in plasmatic prostaglandin E2 (PGE2), regenerating islet-derived protein 4 (Reg IV), epidermal growth factor receptor (EGFR), and (protin kinase B) Akt concentrations. The results suggest that proteoglycan acts as an immunopotentiator partly through protecting T cells from escaping PGE2 attack and enhancing the mucosal IgA response, and as a direct inhibitor by disrupting the Reg IV/EGFR/Akt signaling pathway.

Song et al. (2008) deciphered the anti-cancer effects of *Phellinusigniarius*. The ethanolic extract from the fruiting body of *P*. *igniarius* was used to evaluate the anti-proliferative and anti-metastatic effects in human hepatocarcinoma (SK-Hep-1) and rat heart vascular endothelial (RHE) cells. The extract inhibited the proliferation of both cell lines in a dose-dependent manner, and the IC_50_ values at 48 h were 72 and 103 μg/mL for SK-Hep-1 cells and RHE cells, respectively. Ethanol extract at 25 μg/mL completely inhibited matrigel-induced tube formation in RHE cells. Importantly, the extract at concentration 25 or 50 μg/mL in combination with oxaliplatin (Oxa) or 5-fluorouracil (5-FU) synergistically inhibited the proliferation of SK-Hep-1 cells. These results demonstrate the potential of *P*. *igniarius* ethanol extract as an adjuvant for chemotherapy.

The downregulation of MDM2, the proto-oncogene inhibiting the tumor-suppressor function of p53 is considered an attractive cancer therapeutic strategy (Lu et al. 2009). Hispolon extracted from *Phellinus* species induce epidermoid and gastric cancer-cell apoptosis. Regardless of p53 status, hispolon inhibited breast- and bladder-cancer cell growth. Hispolon treatment elevated p21 concentration, a cyclin-dependent kinase inhibitor and degraded MDM2, a negative regulator of p21 by ubiquitination. Studies indicated that cells with higher ERK1/2 activity were more sensitive to hispolon. Crucial role of hispolon in ubiquitination and downregulation of MDM2 via MDM2-recruited activated ERK1/2 was reported, projecting the phenolic compound as a potential anti-tumor agent in breast and bladder cancers.

### Genus *Pleurotus*

Lavi et al. (2006) reported that an aqueous polysaccharide extract from *Pleurotusostreatus* induces anti-proliferative and pro-apoptotic effects on HT-29 colon cancer cells. A novel water-soluble polysaccharide (POPS-1) was obtained from the fruiting bodies of *P*. *ostreatus* by hot-water extraction, ethanol precipitation, and fractionation by DEAE-cellulose ion exchange and Sepharose CL-6B gel filtration chromatography. Cytotoxicity assay showed POPS-1 presented significantly higher anti-tumor activity against HeLa tumor cell in vitro, in a dose-dependent manner, and exhibited significantly lower cytotoxicity to human embryo kidney 293T cells than HeLa tumor cells compared with anticancer drug 5-fluorouracil. The results suggest POPS-1 may be considered as a potential candidate for developing a novel low-toxicity anti-tumor agent (Tong et al. 2009).

Li et al. (2008a) isolated a homodimeric 32.4 kDa lectin from fresh fruiting bodies of the mushroom*Pleurotuscitrinopileatus*. The lectin exerted potent anti-tumor activity in mice bearing sarcoma 180, and caused approximately 80% inhibition of tumor growth when administered intraperitoneally at 5 mg/kg daily for 20 days.

Wong et al. (2007) studied the in vitro anti-proliferative activities of the water-soluble polysaccharides extracted from the fruiting body and mycelium of a novel edible mushroom*Pleurotustuber*-*regium.* Fruiting body extract showed the strongest cytotoxicity (approximate IC_50_ 25 μg/mL) and exerted effective anti-proliferative activity at 200 μg/mL against human acute promyelocytic leukemia cells (HL-60). Both polysaccharide extracts induced apoptosis in HL-60 cells with an increase in the ratio of Bax/Bcl-2. Analysis from flow cytometry and western blot demonstrated that mycelium extract caused G_2_/M arrest in HL-60 cells by lowering the Cdk1 expression, while fruiting body caused S arrest in the HL-60 cells by a depletion of Cdk2 and an increase in cyclin E expression.

### Genus *Agaricus*

*Agaricusblazei* Murill has been conventionally used as a health food for the prevention of cancer. *Agaricusblazei* Murrill extracts have immunomodulatory, anti-carcinogenic and anti-mutagenic properties as studied by its action on clastogenicity induced by cyclophosphamide (CP) in mice (Delmanto et al. 2001). *Agaricusbisporus* lectin (ABL) and *Agaricuspolytricha* protein (APP) are stable immune stimulants, for health food and pharmaceutical utilization (Chang et al. 2007). In vitro, *A*. *bisporus* extract can suppress aromatase activity and prevent breast-cancer cell proliferation. The broth fraction of *A*. *blazei*, when examined on the growth of human prostate cancer inhibited cell proliferation in both androgen-dependent and androgen-independent prostate cancer cell lines. The broth of *A*. *blazei* induced lactate dehydrogenase leakage in three cancer cell lines, whereas the activities of caspase 3 and the DNA fragmentation were enhanced the most in androgen-independent PC3 cells. The protein expressions of apoptosis-related molecules were elevated by the broth of *A*. *blazei* in PC3 cells. Oral supplementation with the broth of *A*. *blazei* (with the higher ratio of β-glucan) significantly suppressed tumor growth without inducing adverse effects in severe combined immunodeficient mice with PC3 tumor xenograft. Tumor xenografts from *A*. *blazei*-fed mice showed decreased proliferating cell nuclear antigen-positive cells and reduced tumor microvessel density (Yu et al. 2009). Akiyama et al. (2011) studied the effects of agaritine, a hydrazine-derivative from hot-water extract of *A*. *blazei* Murrill on human leukemic monocyte lymphoma (U937) cells. Agaritine induced DNA fragmentation, annexin V expression, and cytochrome c release. Caspase-3, 8, and 9 activities are gradually increased after agaritine treatment. These results suggest that agaritine moderately induces apoptosis in U937 cells. *Agaricusblazei* has been used as an adjuvant in cancer chemotherapy and various types of anti-leukemic bioactive components have been extracted from it. MTT and tritiated thymidine incorporation assays were used to evaluate the in vitro anti-leukemic effects. The most potent extract was further investigated using human promyelocytic leukemia (NB-4) cells-bearing nude mice. Kim et al. (2009) reported that the extract JAB80E70 showed the most potent tumor-selective growth inhibitory activity against human leukemia NB-4 and K-562 cells. DNA fragmentation assays and cell death detection by ELISA showed that the fraction induces apoptosis in NB-4 cells. Adams et al. (2008) evaluated the effects of *A*. *bisporus* extract in vivo and its major component, conjugated linoleic acid on prostate cancer cell lines in vitro, respectively. DU145 and PC3 prostate tumor size and tumor cell proliferation were decreased in nude mice treated with mushroom extract. Microarray analysis of tumors identified significant changes in gene expression in the mushroom-fed mice as compared to controls. The conjugated linoleic acid inhibited proliferation in the prostate cancer cell lines in vitro.

### *Lentinulaedodes*

Shiitake mushrooom,*Lentinulaedodes* produces lentinan, a β-glucan known to suppress leukemia cell proliferation. The ethanol extract of this mushroom significantly decreased cell proliferation of CH72 cells, whereas it could not change the proliferative response of the non-tumorigenic keratinocyte (C50) cell line. Cell cycle analysis demonstrated that *L*. *edodes* extract induced a transient G_1_ arrest, with no changes observed in C50 cells (Gu and Belury 2005)**.**

### *Trametesversicolor* or*Coriolusversicolor*

The turkey tail mushroom or cloud mushroom,*Trametesversicolor* or*Coriolusversicolor* has been studied to have anti-tumor property against many types of cancers. Hsieh and Wu (2001) studied that ethanolic extracts of Yunzhi, a proprietary dietary supplement prepared from extracts of*T*. *versicolor* reduces the growth of hormone responsive prostate cancer LNCaP cell growth. The polysaccharo-peptide in the extract raises the possibility that Yunzhi may be considered as an adjuvant therapy in the treatment of hormone responsive prostate cancer; additionally, it may have chemopreventive potential to restrict prostate tumorigenic progression from the hormone-dependent to the hormone-refractory state. Chu et al. (2002) reviewed that the growth of several human cancer cell lines, viz., gastric cancer (7907), lung cancer (SPC), leukemia (MCL), and lymphoma (SLY)-was markedly inhibited by a crude*C*. *versicolor* extract at 1 mg/mL after 72 h of incubation. The polysaccharide peptide krestin of*T*. *versicolor* has potential to be used as an adjuvant in breast cancer prevention (Standish et al. 2008). The polysaccharide of this mushroom has been demonstrated to inhibit the proliferation of cancer cells in vitro and in vivo, examined on the human hepatoma cancer (QGY) cell lines. These results showed that the polysaccharide inhibited the proliferation in low concentrations (20 mg/L) and the IC50 value was 4.25 mg/L. Apoptosis and significant decrease in the expression of the cell cycle-related genes (*p53, Bcl*-*2,* and *Fas,*) in these cells following treatment, indicate that the polysaccharide can be a potential candidate in cancer therapy (Cai et al. 2010).

### *Grifolafrondosa*

*Grifolafrondosa*, commonly known as the dancing mushroom or Maitake is regarded to impart vitality to health. A β-glucan purified from *G*. *frondosa* enhances the efficacy of anti-cancer agent cisplatin, checking the decrease in the number of immunocompetent cells, viz. macrophages, DCs and NK cells in cisplatin-treated mice (Masuda et al. 2009). A chemically sulfated polysaccharide (S-GAP-P) derived from water-insoluble polysaccharide of *G*. *frondosa* mycelia was investigated for its anti-cancer effects alone and in combination with 5-fluorouracil (5-FU) on human gastric carcinoma (SGC-7901) cells. Results showed that S-GAP-P inhibited SGC-7901 cells growth in a dose-dependent manner and induced cell apoptosis. The combination of S-GAP-P (10–50 μg/mL) with 1 μg/mL 5-FU resulted in a significant inhibition on SGC-7901 cells growth. The results confirm that S-GAP-P has evident anti-cancer activity through apoptotic induction and could significantly accelerate the anti-cancer activity of 5-FU (Shi et al. 2007). Cui et al. (2007) investigated the biological function of a novel polysaccharide-peptide GFPPS1b, isolated from cultured mycelia of *G*. *frondosa* GF9801. GFPS1b has anti-tumor activity which significantly inhibited the proliferation of human gastric adenocarcinoma (SGC-7901 cells), whereas slightly influenced the growth of human normal liver (L-02) cell line. When treated with GFPS1b, SGC-7901 cells succumbed to apoptotis as evidenced from the loss of villus and appearance of apoptotic bodies on the cell surface, volume reduction, and chromatin condensation. The results of flow cytometry analysis and annexin V-PI assay showed that the SGC-7901 cell cycle was arrested in the G_2_/M phase. The apoptotic machinery was associated with drop in mitochondrial trans-membrane potential, up-regulation of Bax, downregulation of Bcl-2, and activation of caspase-3.

### Genus *Ganoderma*

*Ganoderma*, commonly known as Lingzhi or Reishi, also called mushroom of immortality, belonging to family Ganodermataceae has been traditionally administered throughout Asia for centuries as a cancer treatment. *Ganodermalucidum* exhibits anti-cancer effect alone or in combination with chemotherapy and radiotherapy (Pillai et al. 2010). The effects of ethanol extracts of *G*. *lucidum* on the growth of human gastric carcinoma (AGS) cell line were investigated which showed decrease in their viability.The treatment induced the expression of proteins such as death receptor 5 and tumor necrosis factor-related apoptosis-inducing ligand, which further triggered the activation of caspase-8 and the cleavage of Bid. The increase in apoptosis induced by the extract was correlated with activation of caspase-9 and -3, downregulation of IAP family proteins such as XIAP and survivin, and concomitant degradation of poly (ADP-ribose) polymerase. The results indicated that EGL induces the apoptosis of AGS cells through a signaling cascade of death receptor-mediated extrinsic, as well as mitochondria-mediated intrinsic, caspase pathways which are associated with inactivation of the Akt signal pathway (Jang et al. 2010). Chen and Zhong (2011) reported the inhibition of tumor invasion and metastais by ganoderic acid T, a lanostane triterpenoid *G*. *lucidum*. Ganoderic acid T promoted cell aggregation, inhibited cell adhesion, and surpressed cell migration with a dose-dependent manner in human colon tumor cell lines of HCT-116 p53+/+ and p53−/−. GA-Me was found to possess remarkable cytotoxicity on human colon carcinoma (HCT-116) cells in a dose-dependent manner. The expression of anti-tumor protein p53 in GA-Me treated tumor cells was increased in a time dependent manner. Among the pro-apoptotic proteins, Bax was up-regulated, whereas the expression of Bcl-2 was not significantly changed, thus the ratio of Bax/Bcl-2 was increased. Furthermore, GA-Me reduced mitochondrial transmembrane potential, released cytochrome *c* and increased caspase 3 activity during the induced apoptotic process. Our findings show that the anti-cancer bioactivity of GA-Me was mediated by induced apoptosis, resulting from mitochondrial dysfunctions. Zhou et al. (2011) suggested that GA-Me may be a novel promising agent for the treatment of human colon carcinoma cells by mitochondrial pathway manipulation. Liu and Zhong (2010) investigated the effects of a pair of positional isomer of ganoderic acids, ganoderic acid Mf (GA-Mf) and ganoderic acid S (GA-S) on induction of HeLa cells apoptosis. The results demonstrate that both isomers decreased cell population growth on various human carcinoma cell lines by MTT assay, while GA-Mf had better selectivity between normal and cancer cells. Flow cytometry results and cell cycle arresting phases show that, compared with GA-S, GA-Mf was more efficient in inducing apoptosis. Treatment of HeLa cells with each isomer decreased the mitochondrial membrane potential and caused the release of cytochrome *c* from mitochondria into the cytosol, causing stimulation of caspase-3 and caspase-9 activity was observed. The Bax/Bcl-2 ratio was also increased in GA-treated HeLa cells. A native glycopeptide, LZ-D-4 purified from the fruiting bodies of *G*. *lucidum*, and its sulfated derivative, LZ-D showed anti-tumor test in vitro against mouse lymphocytic leukemia (L1210) cell (Ye et al. 2009). The dichloromethane extract *G*. *lucidum* possessing flavonoids, terpenoids, phenolics, and alkaloids has anti-human papillomavirus 16 (HPV 16) E6 oncoprotein activity. Epidermoid cervical carcinoma (CaSki) cells when treated with the crude dichloromethane extracts HPV 16 E6 production was suppressed (Lai et al. 2010). Hsu et al. (2008) studied the anti-tumor effects of*Ganodermatsugae* extracts on colorectal adenocarcinoma cell proliferation. Tumorigenesis study in nude mice revealed the extracts caused tumor shrinkage. In vitro and in vivo experiments showed that colorectal adenocarcinoma cells are inhibited by induction of G_2_/M cell cycle arrest. It may be through downregulation of cyclin A and B1 and up-regulation of p21 and p27. Also, no significant physiological changes as a result of treatment with *G*. *tsugae* extracts were observed in the animal model. Purified recombinant fungal immunomodulatory protein from *G*. *tsugae* (*re*FIP-gts) has anti-telomerase effects in human lung adenocarcinoma (A549) cells. Liao et al. (2008) demonstrated that *re*FIP-gts-treated lung cancer cells undergo premature cellular senescence and are arrested at G1 phase. The *re*FIP-gts- treated A549 cells grew slowly and formed significantly fewer cell colonies.

### Hericium erinaceus

*Hericium erinaceus* or commonly known as Lions mane has attracted great attention owing to its anti-tumor and immunomodulatory effect (Wang et al. 2004). Lee and Hong (2010) demonstrated that *H*. *erinaceus* acts as an enhancer to sensitize doxorubicin (Dox)-mediated apoptotic signaling by reducing c-FLIP expression via JNK activation and enhancing intracellular Dox accumulation via the inhibition of NF-κB activity. Kim et al. (2011) investigated the anti-tumor effects of the extracts of this mushrooms in Balb/c mice transplanted with CT-26 colon cancer cells. The β-glucan rich hot-water obtained by boiling and microwaving, when injected daily for 2 weeks, significantly reduced tumor weights by 38 and 41%, respectively. Tumor regressions were associated with increase in natural killer cells and tumor necrosis factors. The pro-angiogenic factors, including vascular endothelial growth factor (VEGF), cyclooxygenase 2 (COX-2), and 5-lipoxygenase (5-LOX) were also significantly reduced in mRNA and protein expression by tumor genes. Reduced COX-2 and 5-LOX expression further triggered the inhibition of neo-angiogenesis inside the tumors. It was concluded that induction of NK activity, activation of macrophages, and inhibition of angiogenesis all contribute to the mechanism of reduction of tumor size.

### *Cordycepsmilitaris*

Rao et al. (2010) purified bioactive compounds from *Cordyceps militaris* extracts which displayed potent growth inhibition on nitric oxide, tumor necrosis factor-α, and interleukin-12 production from LPS/IFN-γ-stimulated macrophages, along with anti-proliferation effect against human cancer cells, viz. prostate (PC-3), colon 205 and hepatoma (HepG2) cells. Kim et al. (2010) investigated the effects of polysaccharide cordlan isolated from *C*. *militaris* on dendritic cell maturation. Cordlan induce phenotypic maturation of dendritic cells as demonstrated by the elevated expressions of CD40, CD80, CD86, MHC-I and MHC-II molecules. Also, cordlan increased phosphorylation of ERK, p38, and JNK, and nuclear translocation of NF-κB p50/p65, which were main signaling molecules down-stream from TLR4. Defect of dendritic cell maturation in tumor microenvironments is an important immunological problem limiting the success of cancer immunotherapy. *Cordyceps militaris* extracts significantly induced level of IL-18 transcription via enhancing of P1 promoter region in mouse brain and liver and activated the IFN-γ production in mouse leukemic monocyte macrophage cell line (RAW 264.7). The result indicates its potential as an immune activator or anti-cancer drug (Kim et al. 2008). Park et al. (2009) investigated the anti-tumor effect of *C*. *militaris* in NCI-H406 cell-transplanted nude mice. After feeding an aqueous solution of *C*. *militaris* extracts in NCI-H460 cell-xenografted nude mice for 4 weeks, *C*. *militaris* shrunk tumors and increased mouse lifespan, suggesting that *C*. *militaris* was effective in treating tumors in nude mice. Treatment with cordycepin from *C*. *militaris* significantly inhibited human leukemia cell growth in a concentration-dependent manner by inducing apoptosis. This induction was associated with generation of reactive oxygen species (ROS), mitochondrial dysfunction, activation of caspases, and cleavage of poly (ADP-ribose) polymerase protein. Cordycepin induces apoptosis of human leukemia cells through a signaling cascade involving a ROS-mediated caspase pathway (Jeong et al. 2011).

### *Boletusbadius* or*Xerocomusbadius*

Theanine (γ-glutamylethylamide) having anti-tumor activity is produced from *Xerocomusbadius* by submerged fermentation (Li et al. 2008b). l-theanine has synergistic effect on the anti-tumor activities of doxorubicin, anthracyclines, cisplatin, and irinotecan. Consequently, the modulating effect of theanine on the efficacy of anti-tumor agents is expected to be applicable in clinical cancer chemotherapy.

### *Calvatiautriformis*

*Calvatiautriformis* or *Handkeautriformis* or commonly known as puffballs belonging to Lycoperdaceae family has been reported to possess anti-oncogenic properties. Lam et al. (2001) reported high anti-proliferative activity toward breast cancer cells by an ubiquitin-like peptide isolated from *C*. *utriformis* fruit-bodies. Ng et al. (2003) isolated a novel ribosome-inactivating protein calcaelin having translation-inhibiting and anti-mitogenic activities from *C*. *utriformis*. Calcaelin reduced the viability of breast cancer cells. Strong antibiotic activity of calvatic acid and some of its analogs against gastric cancer pathogen *Helicobacterpylori* has been reported (Coetzee and van Wyk 2009).

### Schizophyllum commune

*Schizophyllumcommune* or commonly known as split-gill mushroom belongs to family Schizophyllaceae*.* Schizophyllan, a non-ionic, water-soluble homopolysaccharide consisting of a linear chain of β-d-(1-3)-gluco-pyranosyl groups and β-d-(1-6)-glucopyranosyl groups produced by *S*. *commune* ATCC 38548 has attracted attention in the recent years in pharmaceutical industry as immunomodulatory and anti-neoplastic agent (Kumari et al. 2008).

### *Flammulinavelutipes*

*Flammulinavelutipes* is an edible mushroom, commonly known as winter mushroom, velvet foot or enoki, falling under Physalacriaceae family. A fungal immunomodulatory protein (FIP-fve), an activator of human T lymphocytes purified from *F*. *velutipes* has shown anti-tumor effect on oral administration in murine hepatoma model (Chang et al. 2010). From the aqueous extract of fruit bodies of *F*. *velutipes*, flammulin, an anti-tumor substance, was purified. A stable hemagglutinin was isolated from the fruiting bodies of this mushroom, which inhibits proliferation of leukemia L1210 cells (Ng and Ngai 2006). Water-based extracts of *F*. *velutipes* was identified as novel anti-breast-cancer agents. It could markedly inhibit growth of (estrogen receptor) ER+ (MCF-7) and ER− (MDA-MB-231) breast cancer cells. The extract induced an exceptionally rapid apoptosis on both types of cancer cells. The degree of cytotoxicity on ER− breast cancer cells was very high, whereas the ER− breast cancer cells are inhibited by about 99%, following FVE treatment (Gu and Leonard 2006).

### *Suillusplacidus*

Irofulven or 6-hydroxymethylacylfulvene is a novel semisynthetic anti-tumor agent derived from the sesquiterpene mushroom toxin illudin S of *Suillusplacidus*. Human liver cancer cells (HepG2 cells, Hep3B cells, and SK-Hep-1) were preferentially killed by suillin. Liu et al. (2009) found for the first time that suillin induces apoptosis in HepG2 cells as characterized by DNA fragmentation, phosphatidyl-serine externalization, activation of caspase-3, -8, and -9, depolarization of mitochondrial membrane potential, as well as release of cytochrome *c* into the cytosol. Suillin also causes significant increases in the protein levels of Fas death receptor, adaptor FADD protein, pro-apoptotic protein Bad and a decline of Bid.

### *Inonotusobliquus*

*Inonotusobliquus* (Chaga mushroom) belonging to Aphyllophoromycetodeae, one of the widely known medicinal mushrooms, has been used to treat various cancers in Russia and most of Baltic countries for many centuries. Hot-water and ethanol extract of *I*. *obliquus* has ability to induce apoptosis in human colon cancer (DLD-1) cells by prevention of reactive oxygen species (ROS)-induced tissue damage (Hu et al. 2009). Youn et al. (2009) examined the anti-proliferative effects of water extract of *I*. *obliquus* extract on murine melanoma (B16-F10) cells. The extract not only inhibited the growth of B16-F10 cells by arresting cell cycle at G_0_/G_1_ phase and causing apoptosis, but also induced cell differentiation. These effects were associated with the down-regulation of pRb, p53 and p27 expression levels, and further showed that *I*. *obliquus* extract resulted in a G_0_/G_1_ cell cycle arrest with reduction of cyclin E/D1 and Cdk 2/4 expression levels. Furthermore, the anti-tumor effect of *I*. *obliquus* extract was assessed in vivo in Balb/c mice. Intraperitoneal administration of *I*. *obliquus* extract significantly inhibited the growth of tumor mass in B16-F10 cells implanted mice, resulting in a 3-fold inhibition at dose of 20 mg/kg/day for 10 days. The ethanolic extract of sclerotium and fruiting body of *I*. *obliquus* elicited significant anti-tumor activity, 74.6 and 44.2%, respectively (Yong et al. 2011).

### *Inocybeumbrinella*

Psilocybin or magic mushrooms contain the psycho-active compound psilocybin. Moderate doses (0.2 mg/kg) of the hallucinogen psilocybin treatment demonstrated a significant reduction in anxiety, mood improvement and, spirit lift in patients battling cancer. This study established the feasibility and safety of administering moderate doses of psilocybin to patients with advanced-stage cancer and anxiety (Grob et al. 2011). A novel lectin extracted from the toxic mushroom *Inocybeumbrinella* inhibits proliferation of tumor HepG2 and MCF7 cells (Zhao et al. 2009).

### *Coprinuscomatus*

The shaggy ink cap mushroom, *Coprinuscomatus* belonging to Agaricomycetideae was evaluated for its anti-cancer potential. The IC_50_ value of the mushroom ethyl acetate extract was only 32 μg/mL. The extract significantly affected IκBα phosphorylation in a dose-dependent manner. The effect of ethyl acetate extract was comparable to the effect of curcumin, a known NF-κB pathway inhibitor. Also, the ethyl acetate extract inhibited the activity of IKK complex, at close to 90% as compared to the control of the untreated sample. The results promise that the mushroom extract can be an effective therapy for malignant estrogen-independent breast cancer (Asatiani et al. 2011). Zaidman et al. (2008) studied the selective inhibition of prostate cancer LNCaP cells by ethanol and ethyl acetate extracts of this mushroom. It was observed that this extract inhibits dihydrotestosterone-induced LNCaP cell viability and causes a G1 phase arrest. These findings suggested the therapeutic mechanism of the extract as androgen receptor or non-androgen receptor mediated.

### *Funliatrogii*

Aqueous extract of the mycelia of *Funliatrogii* extract shows good anti-tumor toxicity against a range of tumor cell lines (Rashid et al. 2011). A variety of biological assays were used to show that a 4 h exposure of HT29, LNCaP, PC3, MCF-7 and MDA-MB-231 tumor cells to extract (0.5–5.0 mg/mL) resulted in significant cytotoxicity. In a clonogenic assay, IC_50_ values were found to range from 0.4 to 0.72 mg/mL; exposing fibroblast cells to the extract resulted in no cell death, whereas proliferating endothelial cells were killed. When tumors grown in immune compromised mice were injected intratumourally with extract (5 mg/mL twice a week for two weeks), a 9 day tumor-growth delay was observed.

### *Lactariusflavidulus*

The Japanese mushroom *Lactariusflavidulus* mycelial culture has anti-cancer properties. The polysaccharides extracted from this mushroom when administered intraperitoneally into white mice at a dosage of 300 mg/kg inhibited the growth of Sarcoma 180 by 100%. Wu et al. (2011) isolated a dimeric 29.8-kDa lectin from dried *L*. *flavidulus* fruit bodies. The lectin suppressed proliferation of HepG2 and L1210 cells with an IC_50_ of 8.90 μM and 6.81 μM, respectively.

### Genus *Clitocybe*

Some members of genus *Clitocybe* belonging to Tricholmataceae family have anti-cancer potential. An immunomodulatory protein CNL belonging to the ricin B-like lectin superfamily synthesized by *Clitocybe nebularis* has anti-proliferative effect which appears to be elicited by binding to carbohydrate receptors on human leukemic T cells. CNL also has potential therapeutic applications in treating hematopoietic malignancies (Pohleven et al. 2009). The laccase enzyme from *Clitocybe maxima* exhibited anti-proliferative activity against Hep G2 and MCF-7 tumor cells (Zhang et al. 2010a). The ethanolic extract of *Clitocybealexandri* was found a very potent inhibitor of the growth of lung, breast, colon, and gastric cancer cell lines (Vaz et al. 2010). The ethanolic extract of this mushroom exhibited significant potency against human lung cancer (NCI-H460) cells. It was observed that the extract induced an S-phase cell cycle arrest, increased the percentage of apoptotic cells and enhanced the levels of p53. Cinnamic acid was found to be the most potent compound regarding cell-growth inhibition. It was verified that concomitant use of the extract provided the strongest decrease in viable cell number.

### *Albatrellusconfluens*

*Albatrellusconfluens* is a polypore mushroom belonging to the family Albatrellaceae*.* Grifolin, a secondary metabolite isolated from the fresh fruiting bodies of *A*. *confluens*, has been shown to inhibit the growth of some cancer cell lines in vitro by up-regulating death-associated protein kinase 1 DAPK1 via p53 in nasopharyngeal carcinoma cells (Luo et al. 2011). Ye et al. (2007) identified the novel targets of grifolin by studying its effect on the human nasopharyngeal carcinoma (CNE1) cell line. Following grifolin treatment, a concomitant inhibition of cyclin D1, cyclin E, CDK4 expression, and subsequent reduction in pRB phosphorylation occurred. Meanwhile, grifolin treatment also resulted in a significant up-regulation of CKI (p19^INK4D^). It proves that both the ERK1/2 and the ERK5 pathways are involved in the inhibition and significantly cause cell-cycle arrest in G1 phase. Grifolin induces dephosphorylation and up-regulates death-associated protein kinase 1 (DAPK1) in nasopharyngeal carcinoma cells NPCs and HONE1. Grifolin promoted the protein–protein interaction of DAPK1 and ERK1/2 to prevent ERK1/2 nucleolus translocation. Findings indicate that grifolin might represent a promising candidate in the prevention and intervention of cancer by targeting DAPK1 signaling to induce cell cycle G1 phase arrest (Luo et al. 2011).

### Genus *Russula*

Ergosta-4,6,8(14),22-tetraen-3-one (ergone), a bioactive steroid from *Russulacyanoxantha* has been demonstrated to possess cytotoxic and anti-proliferative activity towards HepG2 cells. Zhao et al. (2011) unraveled the molecular mechanisms behind cytotoxic activity of ergone. HepG2 cells treated with ergone showed typical markers of apoptosis: (a) G2/M cell cycle arrest, (b) chromatin condensation, (c) nuclear fragmentation, and (d) phosphatidylserine exposure. Furthermore, PARP-cleavage; activation of caspase-3, -8, and -9; up-regulation of Bax and down-regulation of Bcl-2 were observed in HepG2 cells treated with ergone. In this study, we reported for the first time that ergone induced apoptosis through activating the caspase. These results would be useful for the further utilization of many medicinal fungi in cancer treatment. A lectin isolated from *Russula lepida e*xhibited anti-proliferative activity toward Hep G2 cells and MCF-7 cells with an IC50 of 1.6 μM and 0.9 μM, respectively. Daily intraperitoneal injections of the lectin (5.0 mg/kg) for 20 days brought about 67.6% reductions in the weight of S-180 tumor (Zhang et al. 2010b).

### *Fomesfomentarius*

Chen et al. (2011) studied that ethanol extract of mycelial biomass and intracellular polysaccharide of *Fomes fomentarius* play crucial roles in gastric cancer intervention. Both the extracts exhibit anti-proliferative effect on human gastric cancer cell lines SGC-7901 and MKN-45 in a dose-dependent manner. In contrast, human normal gastric cell line GES-1 was less susceptible to EEM and IPS. These results suggest that *F*. *fomentarius* may represent a promising novel approach for gastric cancer intervention. Furthermore, the exopolysaccharide from this mushroom has a direct anti-proliferative effect in vitro on SGC-7901 cells in a dose- and time-dependent manner. Also, this exopolysaccharide sensitized doxorubicin (Dox) and induced growth inhibition of SGC-7901 cells at noncytotoxic concentration of 0.25 mg/mL after 24 h treatment (Chen et al. 2008).

### *Piptoporusbetulinus*

*Piptoporusbetulinus*, commonly known as the birch polypore belonging to Fomitopsidaceae family has been studied in vitro for its anti-cancer activity. The fraction prepared from dried fruiting bodies was subjected to anti-cancer evaluation in human lung carcinoma (A549), colon adenocarcinoma (HT-29) and rat glioma (C6) cell cultures. *P*. *betulinus* fraction elicited anti-cancer effects that were attributed to decreased tumor cell proliferation, motility and the induction of morphological changes. Moreover, it produced no or low toxicity in tested normal cells (Lemieszek et al. 2009).

### Genus *Antrodia*

*Antrodia* (camphor tree mushroom) is a genus of mushrooms in the famly Fomitopsidaceae. These mushrooms are highly valued in Taiwan. The fermented culture broth of *Antrodiacamphorata* has been shown to promote cell cycle arrest and apoptosis of human estrogen-non-responsive breast cancer (MDA-MB-231) cells. Yang et al. (2011) demonstrated that non-cytotoxic concentrations (20–80 μg/mL) of *A*. *camphorata* markedly inhibit the invasion/migration of highly metastatic MDA-MB-231 cells through suppression of the MAPK signaling pathway. Antroquinonol, a ubiquinone derivative isolated from *A*. *camphorata*, induced a concentration-dependent inhibition of cell proliferation in pancreatic cancer (PANC-1 and AsPC-1) cells. Flow cytometric analysis showed that antroquinonol induced G1 arrest of the cell cycle and a subsequent apoptosis. Antroquinonol induces anti-cancer activity in human pancreatic cancers through an inhibitory effect on PI3-kinase/Akt/mTOR pathways that in turn down-regulates cell cycle regulators. The translational inhibition causes G1 arrest of the cell cycle and an ultimate mitochondria-dependent apoptosis. Antroquinonol also induced the cross talk between apoptosis, autophagic cell death and accelerated senescence in cancer cells (Yu et al. 2011). The solid-state extracts of *A*. *camphorata*, when combined with anti-tumor agents showed adjuvant anti-proliferative effects on hepatoma (C3A and PLC/PRF/5) cells and on xenografted cells in tumor-implanted nude mice, extending their median survival days. The inhibition effect was elucidated to be through intervention of MDR gene expressions and COX-2- dependent inhibition of p-AKT (Chang et al. 2008). Peng et al. (2007) studied that the *A*. *camphorata* crude extract has significant suppressive effects on the growth and proliferation of the transitional cell carcinomas cell lines, the superficial cancer cell line RT4, and metastatic cell lines, TSGH-8301 and T24. On treatment with the extract at 100 μg/mL, the p53-independent overexpression of p21 with simultaneous down alteration of pRb was observed in RT4. On the contrary, treatment with the extract, at 50 μg/mL, result in simultaneous down-regulations of Cdc2 and Cyclin B1, with suppression of the absolute migrating capability of the two cell lines TSGH-8301 and T24, and eventually the cell deaths. A HPLC fraction of alcohol extract isolated from *A*. *camphorata* induced apoptosis in A549 cell by decreasing the expression level of four tumor-related genes, e.g., calpain 1/2 small subunit, galectin-1, Rho GDP inhibitor α and eukaryotic translation initiation factor 5A. Apoptosis is triggered by the mitochondrial pathway and endothelium reticulum stress. Fr-6 also could decrease the production level of eukaryotic translation initiation factor 5A, which is a potential cancer intervention target. Chan et al. (2010) suggested that the anti-cancer activity of *A*. *camphorata* might be due to multiple active metabolites, which work together to induce cell apoptosis via various pathways. Hseu et al. (2007) investigated the fermented culture broth of *A*. *camphorata* to induce apoptosis and inhibit cyclooxygenase-2 (COX-2) in MDA-MB-231 cancer cells. Treatment of these cells with *A*. *camphorata* (40–240 μg/mL) resulted in dose and time-dependent apoptosis, as evidenced by loss of cell viability, chromatin condensation, and internucleosomal DNA fragmentation. Apoptosis in the MDA-MB-231 cells was accompanied by release of cytochrome *c*, activation of caspase-3, -8, and -9, and specific proteolytic cleavage of poly (ADP-ribose) polymerase (PARP). Also, *A*. *camphorata* treatment inhibited COX-2 protein expression and prostaglandin E2 (PGE2) production in MDA-MB-231 cells. Fermented culture broth of *A*. *camphorata* has been shown to induce apoptosis in MDA-MB-231 cells in vitro and in vivo in nude mice. *Antrodiacamphorata* treatment decreased the proliferation of canerous cells by arresting progression through the G1 phase of the cell cycle. Hseu et al. (2008) studied that cell cycle blockade in *A*. *camphorate*-treated MDA-MB-231 cells was associated with reductions in cyclin D1, cyclin E, CDK4, cyclin A, and proliferating cell nuclear antigen (PCNA), and increased CDK inhibitor p27/KIP and p21 in a dose- and time-dependent manner. 5′AMP-activated protein kinase (AMPK) and the mammalian target of rapamycin (mTOR) are two serine/threonine protein kinases and potential targets for cancer chemotherapy against hepatocellular carcinoma cells. Chiang et al. (2010) studied that *A*. *camphorate* extract displayed effective anti-cancer activity against both hepatitis B virus (HBV) DNA-positive and -negative hepatocellular carcinoma cell lines (HepG2, HepG2.2.15, Mahlavu, PLC/PRF/5, SK-Hep1 and Hep3B). Antroquinonol completely abolished cell-cycle progression and caused a subsequent apoptosis. The loss of mitochondrial membrane potential and depletion of mitochondrial content indicated the mitochondrial stress caused by antroquinonol. Antroquinonol displays anti-cancer activity against the hepato carcinoma cells through AMPK activation and inhibition of mTOR translational pathway, leading to G1 arrest of the cell-cycle and subsequent cell apoptosis. Kumar et al. (2011) reported that antroquinonol treatment significantly reduced the proliferation of A549 as evidenced from cell shrinkage, apoptotic vacuoles, pore formation, TUNEL positive cells and increased Sub-G1 cell population with respect to time and dose dependent manner. Antroquinonol-induced apoptosis was associated with disrupted mitochondrial membrane potential and activation of caspase 3 and PARP cleavage in A549 cells. Moreover, antroquinonol treatment down-regulated the expression of apoptosis regulatory proteins Bcl2, which was correlated with the decreased PI3K and mTOR protein levels without altering pro apoptotic and anti apoptotic proteins. Antroquinonol altered the expression level of miRNAs compared with untreated control in A549 cells. The data collectively suggested the anti-proliferative effect of antroquinonol may be a promising chemotherapeutic agent for lung cancer. Tsai et al. (2010) studied that methylantcinate A (MAA), an ergostane type triterpenoid isolated from *A*. *camphorata* inhibited the growth of oral cancer (OEC-M1 and OC-2) cell lines in a dose-dependent manner, without showing cytotoxicity to normal oral gingival fibroblast cells. The mechanism of growth inhibition was apoptosis induction, resultant of caspase-3 activation and DNA fragmentation. Hsu et al. (2007) reported the anti-invasive effect of ethyl acetate extract from *Antrodiacinnamomea* fruiting bodies in the human liver cancer (PLC/PRF/5) cell line. This effect was strongly associated with a concomitant decrease in either the level or activity of VEGF, matrix metalloproteinases (MMP-2, MMP-9 and MT1-MMP), and an increase in the expression of tissue inhibitor of metalloproteinase (TIMP-1 and TIMP-2). The extract inhibited constitutively activated and inducible NF-κB in both its DNA-binding activity and transcriptional activity, also inhibited the TNF-α-activated NF-κB-dependent reporter gene expression of MMP-9 and VEGF. Angiogenesis assay showed that the extract also exhibited an inhibitory effect on angiogenesis.

### *Polyozellusmultiplex*

A Korean wild mushroom *Polyozellusmultiplex* checks cell proliferation in stomach cancer by increased expression of p53 proteins (Lee and Nishikawa 2003). Polyozellin isolated from this mushroom induces phase 2 detoxifying enzymes with cancer-preventive potential in mouse hepatoma cells. Also, it significantly induced differentiation in human myeloid leukaemic cell lines (Kim et al. 2004b).

## Current scenario and future perspectives

Mushroom products are set to create a revolution in therapeutic strategies in curbing various forms of cancers. Several companies are dedicated to prepare anti-cancer formulae from mushroom extracts using state-of-the-art technology and their products are gradually being recognized globally. Zhejiang Fangge Pharmaceutical & Healthcare Products Co. Ltd., a large pharmaceutical company in China specializes in development and marketing of mushroom extracts for anti-cancer uses. The enterprise manufactures and exports polysaccharides from mushrooms *G*. *frondosa*, *L*. *edodes*, *G*. *lucidum*, *A*. *blazei*, *Cordyceps sinensis* and *H*. *erinaceus*. FineCo Ltd., an emerging Korean company develops effective drugs, including anti-cancer formulations from medicinal mushrooms. Its products are now sold in Japan, Hong Kong, Australia, America, and Europe, for use in clinical test on cancer patients. A pioneering mushroom company Mushroom Wisdom, based in USA is engaged in formulating supplements for inhibiting cancer. USA based Aloha medicinal Inc. manufactures an array of medicinal mushroom products including the cancer-inhibiting *Ganoderma* capsules. In near future, more companies are expected to join the flourishing market of cancer therapeutic manufacture from mushrooms. Better insight into the mechanisms underlying biological action of mushroom will accelerate commercial production of pharmaceuticals for cancer therapy.

## Conclusions

Medicinal mushrooms represent a growing segment of today’s pharmaceutical industry owing to the plethora of useful bioactive compounds. While they have a long history of use across diverse cultures, they are backed up by reasonable scientific investigation now. The mycologists around the world, firmly believe that a greater knowledge of mushroom can ameliorate many forms of cancers at various stages. Exploration of unique species with medicinal properties from the untapped wilderness is warranted. Conservation and cloning of therapeutic mushrooms is needed for sustainable development. Dedicated research should be undertaken to isolate, purify and structural investigation of novel anti-cancer and immune-stimulator compounds. Studies to date have identified a number of compounds and elucidated underlying mechanism. However, research is needed to elucidate the different roles of multiple active compounds and the pathways involved. These findings are important due to the lack of chemotherapeutic agents of some forms of malignant cancer, viz. estrogen receptor negative human breast cancer, mesothelioma, acute lymphocytic leukemia, acute myeloid leukemia, Hodgkin lymphoma, hopeless astrocytoma, etc. The present results and data might provide new insights into the possible therapeutic uses of mushrooms and helpful suggestions for the design of anti-tumor drugs from mushrooms in combating cancer.
